# Supplementary material for: MSGene: a multistate model using genetic risk and the electronic health record applied to lifetime risk of coronary artery disease
Source: Nat Commun. 2024 Jun 7;15:4884. doi: 10.1038/s41467-024-49296-9 (PMC11161589; doi:10.1038/s41467-024-49296-9)
Supplement: Supplementary file 3 — Description of Additional Supplementary Files [file 41467_2024_49296_MOESM3_ESM.pdf]

Description of Additional Supplementary Files

File Name: Supplementary Data 1

Description: Predicted Remaining Lifetime Risk of Individuals

Beginning from each state at age 40. Rows represent sex and PRS SD interaction. Standard deviations ascertained after 1000 bootsrapping iterations on the training data.

PRS: Polygenic Risk Score

HT: hypertension

HL: Hyperlipidemia

DM: Diabetes Mellitus (1 or 2)

File Name: Supplementary Data 2

Description: Predicted Remaining Lifetime Risk under statin therapy of Individuals

Beginning from each state at age 40

Rows represent sex and PRS SD interaction. Standard deviations were ascertained after 100 bootstrapping iterations on the training data.

PRS: Polygenic Risk Score

HT: hypertension

HL: Hyperlipidemia

DM: Diabetes Mellitus (1 or 2)

File Name: Supplementary Data 3

Description: Predicted Remaining Lifetime Risk of Individuals

Beginning from each state at age 50. Rows represent sex and PRS SD interaction. Standard deviations ascertained after 1000 bootsrapping iterations on the training data.

PRS: Polygenic Risk Score

HT: hypertension

HL: Hyperlipidemia

DM: Diabetes Mellitus (1 or 2)

File Name: Supplementary Data 4

Description: Predicted Remaining Lifetime Risk under statin therapy of Individuals Beginning from each state at age 50

Rows represent sex and PRS SD interaction. Standard deviations were ascertained after 1000 bootstrapping iterations on the training data.

PRS: Polygenic Risk Score

HT: hypertension

HL: Hyperlipidemia

DM: Diabetes Mellitus (1 or 2)

File Name: Supplementary Data 5

Description: Predicted Remaining Lifetime Risk for Individuals Beginning from each state at age 60

Rows represent sex and PRS SD interaction. Standard deviations were ascertained after 1000 bootstrapping iterations on the training data.

PRS: Polygenic Risk Score

HT: hypertension

HL: Hyperlipidemia

DM: Diabetes Mellitus (1 or 2)

File Name: Supplementary Data 6

Description: Predicted Remaining Lifetime Risk for Individuals under statin therapy Beginning from each state at age 60

Rows represent sex and PRS SD interaction. Standard deviations were ascertained after 1000 bootstrapping iterations on the training data.

PRS: Polygenic Risk Score

HT: hypertension

HL: Hyperlipidemia

DM: Diabetes Mellitus (1 or 2)

File Name: Supplementary Data 7

Description: Predicted Remaining Lifetime Risk for Individuals Beginning from each state at age 70

Rows represent sex and PRS SD interaction. Standard deviations were ascertained after 1000 bootstrapping iterations on the training data.

PRS: Polygenic Risk Score

HT: hypertension

HL: Hyperlipidemia

DM: Diabetes Mellitus (1 or 2)

File Name: Supplementary Data 8

Description: Predicted Remaining Lifetime Risk for Individuals under statin therapy Beginning from each state at age 70

Rows represent sex and PRS SD interaction. Standard deviations were ascertained after 1000 bootstrapping iterations on the training data.

PRS: Polygenic Risk Score

HT: hypertension

HL: Hyperlipidemia

DM: Diabetes Mellitus (1 or 2)

File Name: Supplementary Data 9

Description: Predicted Ten-year Risk of Individuals

Beginning from each state at age 40. Rows represent sex and PRS SD interaction. Standard deviations ascertained after 1000 bootsrapping iterations on the training data.

PRS: Polygenic Risk Score

HT: hypertension

HL: Hyperlipidemia

DM: Diabetes Mellitus (1 or 2)

File Name: Supplementary Data 10

Description: Predicted Ten-year Risk under statin therapy of Individuals
Beginning from each state at age 40
Rows represent sex and PRS SD interaction. Standard deviations were ascertained after 100 bootstrapping iterations on the training data.
PRS: Polygenic Risk Score
HT: hypertension
HL: Hyperlipidemia
DM: Diabetes Mellitus (1 or 2)

File Name: Supplementary Data 11

Description: Predicted Ten-year Risk of Individuals
Beginning from each state at age 50. Rows represent sex and PRS SD interaction. Standard deviations ascertained after 1000 bootsrapping iterations on the training data.

PRS: Polygenic Risk Score
HT: hypertension
HL: Hyperlipidemia
DM: Diabetes Mellitus (1 or 2)

File Name: Supplementary Data 12

Description: Predicted Ten-year Risk under statin therapy of Individuals Beginning from each state at age 50
Rows represent sex and PRS SD interaction. Standard deviations were ascertained after 1000 bootstrapping iterations on the training data.
PRS: Polygenic Risk Score
HT: hypertension
HL: Hyperlipidemia
DM: Diabetes Mellitus (1 or 2)

File Name: Supplementary Data 13

Description: Predicted Ten-year Risk for Individuals Beginning from each state at age 60

Rows represent sex and PRS SD interaction. Standard deviations were ascertained after 1000 bootstrapping iterations on the training data.

PRS: Polygenic Risk Score
HT: hypertension
HL: Hyperlipidemia
DM: Diabetes Mellitus (1 or 2)

File Name: Supplementary Data 14
Description: Predicted Ten-year Risk for Individuals under statin therapy Beginning from each state at age 60
Rows represent sex and PRS SD interaction. Standard deviations were ascertained after 1000 bootstrapping iterations on the training data.

PRS: Polygenic Risk Score
HT: hypertension
HL: Hyperlipidemia
DM: Diabetes Mellitus (1 or 2)

File Name: Supplementary Data 15
Description: Predicted Ten-year Risk for Individuals Beginning from each state at age 70

Rows represent sex and PRS SD interaction. Standard deviations were ascertained after 1000 bootstrapping iterations on the training data.

PRS: Polygenic Risk Score
HT: hypertension
HL: Hyperlipidemia
DM: Diabetes Mellitus (1 or 2)

File Name: Supplementary Data 16
Description: Predicted Ten-year Risk for Individuals under statin therapy Beginning from each state at age 70

Rows represent sex and PRS SD interaction. Standard deviations were ascertained after 1000 bootstrapping iterations on the training data.

PRS: Polygenic Risk Score
HT: hypertension
HL: Hyperlipidemia
DM: Diabetes Mellitus (1 or 2)

File Name: Supplementary Data 17
Description: Ethnicity specific results. We perform the analyses described in Figure 4 on subsets of the UKB. EUR: European, AFR: African, EAS: East Asian, SAS, Southeast Asian

File Name: Supplementary Data 18
Description: Phenotype codes. We report the specific phenotype codes used to define our phenotypes of interest, also available at https://github.com/niekverw/ukbpheno/blob/master/inst/extdata/definitions_cardiometabolic_traits.tsv.
Ht: Hypertension, Dm: Diabetes Mellitus, Hyperlip: Hyperlipidemia, Cad: Coronary Artery Disease.
