# Supplementary material for: MSGene: a multistate model using genetic risk and the electronic health record applied to lifetime risk of coronary artery disease
Source: Nat Commun. 2024 Jun 7;15:4884. doi: 10.1038/s41467-024-49296-9 (PMC11161589; doi:10.1038/s41467-024-49296-9)
Supplement: Supplementary file 2 — Peer Review File [file 41467_2024_49296_MOESM2_ESM.pdf]

nature portfolio

Peer Review File

MSGene: A multistate model using genetic risk and the electronic health record applied to lifetime risk of Coronary artery disease

Open Access This file is licensed under a Creative Commons Attribution 4.0 International License, which permits use, sharing, adaptation, distribution and reproduction in any medium or format, as long as you give appropriate credit to the original author(s) and the source, provide a link to the Creative Commons license, and indicate if changes were made. In the cases where the authors are anonymous, such as is the case for the reports of anonymous peer reviewers, author attribution should be to 'Anonymous Referee' followed by a clear attribution to the source work. The images or other third party material in this file are included in the article's Creative Commons license, unless indicated otherwise in a credit line to the material. If material is not included in the article's Creative Commons license and your intended use is not permitted by statutory regulation or exceeds the permitted use, you will need to obtain permission directly from the copyright holder. To view a copy of this license, visit http://creativecommons.org/licenses/by/4.0/.

Editorial Note: This manuscript has been previously reviewed at another journal that is not operating a transparent peer review scheme. This document only contains reviewer comments and rebuttal letters for versions considered at *Nature Communications.*

Reviewer #1 (Remarks to the Author):

My comments from the prior version have adequately been addressed.

Reviewer #2 (Remarks to the Author):

Thank you for addressing all points raised, I have no further comments.

Reviewer #3 (Remarks to the Author):

The authors were responsive the the reviewers comments and have revised the paper. It remains a very dense and statistically sophisticated paper. The clinical implications and utility remain unclear given this complexity but there are important advances that come from this model.
